# The Mucoid Switch in *Pseudomonas aeruginosa* Represses Quorum Sensing Systems and Leads to Complex Changes to Stationary Phase Virulence Factor Regulation

**DOI:** 10.1371/journal.pone.0096166

**Published:** 2014-05-22

**Authors:** Ben Ryall, Marta Carrara, James E. A. Zlosnik, Volker Behrends, Xiaoyun Lee, Zhen Wong, Kathryn E. Lougheed, Huw D. Williams

**Affiliations:** 1 Department of Life Sciences, Faculty of Natural Sciences, Imperial College London, Sir Alexander Fleming Building, London, United Kingdom; 2 Department of Surgery and Cancer, Faculty of Medicine, Imperial College London, Sir Alexander Fleming Building, London, United Kingdom; University of Rochester, United States of America

## Abstract

The opportunistic pathogen *Pseudomonas aeruginosa* chronically infects the airways of Cystic Fibrosis (CF) patients during which it adapts and undergoes clonal expansion within the lung. It commonly acquires inactivating mutations of the anti-sigma factor MucA leading to a mucoid phenotype, caused by excessive production of the extracellular polysaccharide alginate that is associated with a decline in lung function. Alginate production is believed to be the key benefit of *mucA* mutations to the bacterium in the CF lung. A phenotypic and gene expression characterisation of the stationary phase physiology of *mucA22* mutants demonstrated complex and subtle changes in virulence factor production, including cyanide and pyocyanin, that results in their down-regulation upon entry into stationary phase but, (and in contrast to wildtype strains) continued production in prolonged stationary phase. These findings may have consequences for chronic infection if mucoid *P. aeruginosa* were to continue to make virulence factors under non-growing conditions during infection. These changes resulted in part from a severe down-regulation of both AHL-and AQ (PQS)-dependent quorum sensing systems. *In trans* expression of the cAMP-dependent transcription factor Vfr restored both quorum sensing defects and virulence factor production in early stationary phase. Our findings have implications for understanding the evolution of *P. aeruginosa* during CF lung infection and it demonstrates that *mucA22* mutation provides a second mechanism, in addition to the commonly occurring *lasR* mutations, of down-regulating quorum sensing during chronic infection this may provide a selection pressure for the mucoid switch in the CF lung.

## Introduction

*Pseudomonas aeruginosa* forms intractable, chronic infections in around 80% of Cystic Fibrosis (CF) sufferers and is associated with decreased lung function and an increased risk of respiratory failure and death [1], [2]. It's success as a CF lung pathogen is aided by the expression of a range of virulence factors including the potent toxin hydrogen cyanide, which is regulated by quorum sensing and expressed maximally under low oxygen tension, a condition that *P. aeruginosa* is believed to experience in the thickened mucus of the CF lung [3], [4]. *P. aeruginosa* cyanide production is the mediating factor in the paralytic killing of *Caenorhabditis elegans* [5], is toxic to *Drosophila melanogaster* [6] and cyanide has been detected in burn wound infections caused by *P. aeruginosa* [7]. The potential clinical significance of cyanide in CF lung infections was recently demonstrated by the detection of cyanide in sputum from cystic fibrosis patients infected with *P. aeruginosa* and its presence is associated with a decline in lung function [8], [9]. However, the clinical significance of cyanogenesis and its consequences for the host remain unclear [10], [11]. Cyanide is volatile and its usefulness as a surrogate marker for the diagnosis of *P. aeruginosa* and *Bcc* infection in children who cannot expectorate sputum is currently being investigated [12], [13]. Cyanide has the potential to inhibit aerobic and anaerobic respiration [16]. *P. aeruginosa* avoids the toxic effects of cyanide in part by synthesising a respiratory chain terminated by a cyanide insensitive terminal oxidase [14], [15] and by the action of detoxification mechanisms [16], [17].

A major event in the course of chronic *P. aeruginosa* lung infection is a switch of infecting strains to an alginate over-producing, mucoid phenotype that is often associated with a poor prognosis for the patient [2], [18]. Understanding the consequences of the switch to alginate overproduction is important to an understanding of how *P. aeruginosa* is able to achieve such persistent and problematic CF infections. The mucoid switch most commonly results from loss of regulation of the alternate sigma factor AlgU via mutation in the anti-sigma factor *mucA* [2], [19]. The *P. aeruginosa* MucA protein along with MucB sequesters AlgU to the cytoplasmic membrane. Loss of function mutations of *mucA* result in AlgU being constitutively free to interact with core RNA polymerase and direct transcription from its target genes, which include the alginate biosynthetic genes located in the *algD* operon as well as AlgU's own operon [20], [21], leading to alginate over production and elevated levels of AlgU. One of the most common mutations in *mucA* in CF clinical isolates is the *mucA22* mutation, which results in the truncation of MucA [20], [21].

It is believed that alginate overproduction *per se* provides a major benefit to *P. aeruginosa* of the mucoid switch in the CF lung, due to it providing the bacterium with protection from the host immune response by inhibiting phagocytosis, protecting it against the oxidative burst and providing an immunomodulatory role [22], [23]. However, AlgU regulates genes in addition to the alginate biosynthetic genes, including other transcriptional regulators, such as *algR* and the heat shock sigma factor *rpoH* [24], [25]. Previous studies have shown an increase in type III secretion systems, a decrease in flagellum production and the decrease in C4-HSL-dependent quorum sensing in biofilms are associated with *mucA* mutation [26]–[28]. Recently it was shown that the *mucA22* mutation also affects the osmotic stress sensitivity of *P. aeruginosa* but only in stationary phase, suggesting that *mucA22*, in addition to affecting alginate production during growth, affects cellular physiology in non-growing cells, a factor that could be significant during chronic infections [29], [30].

Given the evidence for the presence of cyanide in the CF lung together with its stationary phase synthesis and the chronic nature of *P. aeruginosa* CF lung infection, our aim was to investigate the effect of the switch to mucoidy on cyanide production by *P. aeruginosa*. We show here that *mucA* mutation causes a more complex and subtle change in cyanide and pyocyanin production than previously recognised, resulting in down-regulation upon entry into early stationary phase and, in contrast to wildtype strains, continued production in prolonged stationary phase. We further show that *mucA* mutation is associated with a profound down regulation of the three major quorum sensing systems of *P. aeruginosa*. We argue that these findings have significant implications in terms of chronic CF lung infection.

## Materials and Methods

### Bacterial strains and growth conditions

The bacterial strains used are listed in Table S1. Overnight starter cultures were prepared by inoculating several colonies into 5 ml LB broth in a 25 ml sterile universal bottle at 37°C with shaking at 200 rpm. For all assays and RNA extractions strains were grown by inoculating 1 ml of overnight culture into 50 ml LB broth in 250 ml conical flasks incubated in an orbital shaker at 200 rpm and 37°C.

### Construction of *mucA22* mutants

Unmarked *mucA22* mutants (loss of a single base) were made in two *P. aeruginosa* wild type backgrounds: PAO1 and PAO381 by gene replacement of the wildtype allele as described previously [29], with the presence of the correct mutation checked by sequencing of PCR amplicons of the putative *mucA22* mutants. Non-mucoid suppressor strains were isolated from *mucA22* strains by growing on LBA plates for several days at 37°C, after which time non-mucoid outgrowths appeared from the edge of the mucoid colony. Suppressor strains are denoted by adding an S to the end of the name of the *mucA22* mutant they were isolated from.

### Cyanide, pyocyanin and elastase assays

Cyanide measurement was performed using a cyanide ion-selective micro-electrode (Lazar Research Laboratories, L.A., CA, USA) as described in [31] and elastase was assayed using the elastin-congo red method of Ohman et al [32]. Pyocyanin was assayed as described by Essar et al [33]. Briefly, the culture supernatant was extracted into chloroform, which was then acidified with 0.2M HCl and the pyocyanin-containing acidic, aqueous layer was removed and it's absorbance determining at 520 nm. The A520 reading was then normalised by dividing by the final OD600 reading of the culture.

### Quorum sensing signal molecule bioluminescent assay

*lux*-reporter strains were used to assay the levels of quorum sensing signal molecules in culture supernatants. Plasmid pSB401 in *E. coli* S17.1 was used for assaying 2-oxo-C12-HSL, plasmid pSB536 in *E. coli* S17.1 was used for assaying C4-HSL and PAO1Δ*pqsA:pqsA::CTXlux* [34], [35] was used for assaying PQS. Luminescence and OD was measured in a FLUOstar Omega plate Reader (BMG Labtech). The luminescence output for each well was blank corrected and normalised to OD. Concentrations of 2-oxo-C12HSL and C4HSL were calculated with reference to an AHL standard curve. PQS levels were calculated relative to PAO1 8 hour supernatant levels.

### qRT-PCR

RNA was extracted using RNeasy Protect Bacteria Mini Kit (Qiagen) using enzymatic lysis and proteinase K digestion with on column DNAase treatment. qRT-PCR was carried out using Rotor-Gene SYBR Green PCR Kit (Qiagen) in a Rotor-Gene 3000 quantitative PCR machine (Corbett). Expression of the target genes was normalised to the expression of 16S RNA.

## Results

### *mucA22* mutants and isolation and use of spontaneous suppressor strains

Isogenic *mucA22* mutants were constructed in PAO1 and PAO381 backgrounds and spontaneous mucoid suppressor strains were isolated from each of the *mucA22* mutants as described in Materials and Methods. We used these as control strains in this study as we wanted to know whether suppression of mucoidy affected key phenotypes associated with *mucA* mutation, particularly as suppressors are likely to accumulate in the CF lung [36]. We did not complement or repair the *mucA22* mutations as a control, because the high frequency of spontaneous mucoid to non-mucoid conversion in our hands made it difficult to be confident that a change to non-mucoidy did not result from the acquisition of a secondary mutation. By gene sequencing we confirmed that the suppressor strains, for which data is reported here, did not have a *mucA22* reverting mutation and neither did they have mutations in *algU*, *algR*, *algD2*, or *lasR*.

### *P. aeruginosa mucA22* strains are deficient in cyanide, pyocyanin and elastase production in early stationary phase

As an initial test cyanide levels were measured in 13 independently isolated *mucA22* strains 2 hours after entry of the cultures into stationary phase. Each of the *mucA22* strains had markedly reduced culture cyanide levels compared to their isogenic wildtype strain equating to an approximate 75% reduction in peak cyanide levels (comparing mean cyanide concentration for each *mucA22* strain to their respective wild type, t-test, all p values<0.001; Fig. S1A). All spontaneous non-mucoid suppressor strains isolated from *mucA22* strains showed a return to wildtype cyanide production. PAO579, a mucoid PAO381 derivative that has made the switch to mucoidy by an *algU/mucA22* independent mechanism [37], produces ∼3 times the cyanide levels of the PAO381 *mucA22* strains (data not shown), strongly suggesting that the phenotype observed is a consequence of *mucA22* mutation rather than alginate over production *per se*. At 2 h into stationary phase the *mucA22* strains produced significantly less pyocyanin than their wild type parents (t-test p<0.001 for each pair; Fig. S1B). In contrast, the suppressor strains showed wild type pyocyanin production. In stationary phase *mucA22* mutants were also defective in elastase production (p<0.01 for each pair) and this phenotype was partially reversed in suppressor strain (Fig. S1C).

### *P. aeruginosa mucA22* strains maintain cyanide and pyocyanin production in late stationary phase

**Figure 1:**
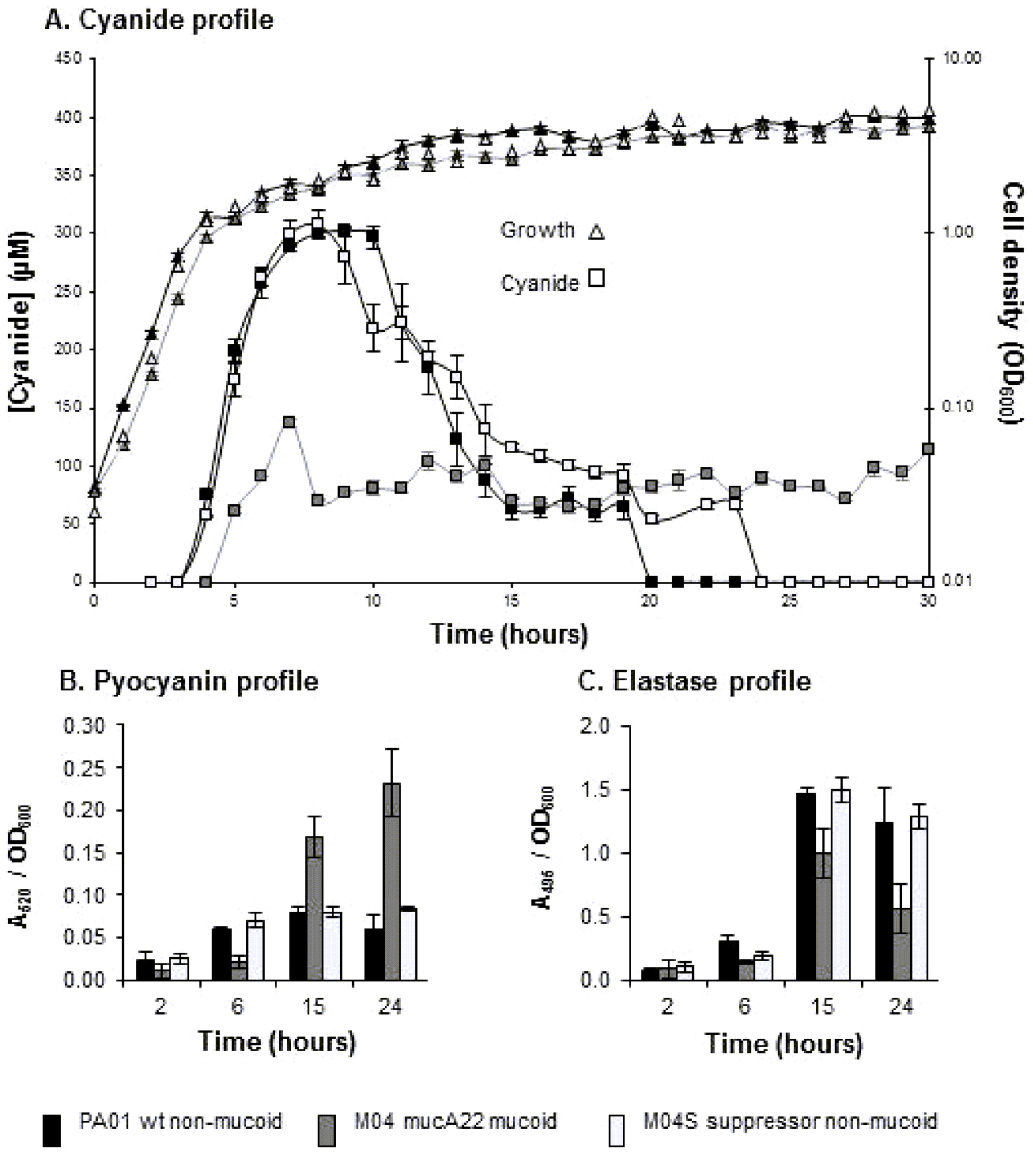
*mucA22*, mucoid strains show differential regulation of virulence factor production throughout grow. **A**, Change in cyanide concentration in liquid culture supernatant (primary y axis, square markers) throughout growth (secondary y axis, triangle markers) for PAO1 (wild type, non-mucoid) (black); M04 (*mucA22*, mucoid) (dark grey); and M04S (non-mucoid suppressor isolated from M04) (light grey). **B** and **C** respectively, pyocyanin and elastase levels in culture supernatant normalised to OD_600_ of culture at 2 hours (mid-exponential phase), 6 hours (early stationary phase),15 hours (mid stationary phase) and 24 hours (late stationary phase). Values are means of 3 independent replicates with SE error bars. Culture conditions and cyanide, pyocyanin and elastase assays were as described in Figure 1.

Investigating the kinetics of cyanide production throughout growth indicated marked differences between wildtype and *mucA22* mutants (Fig. 1A; Fig. S2). For the wild type strain PAO1, cyanide concentrations in culture media increased rapidly at the onset of stationary phase and reached a maximum of ∼300 µM after 8–9 hours in culture, followed by a steady decrease in levels until cyanide was no longer detectable after 20 hours. In contrast, the *mucA22* mutant (M04) had the expected lower peak-level of ∼100 µM cyanide, but these levels were maintained into late stationary phase. The suppressor strains behave similarly to PAO1 and a similar pattern of cyanide production was found in *mucA22* mutants isolated in PAO381 background (Fig. S2). Culture pH was similar for all strains throughout growth, indicating that cyanide gas formation, was not responsible for the differential levels of solution cyanide. We conclude that the wildtype strains switch off cyanide production a few hours into stationary phase and any remaining cyanide is lost from the culture, presumably by blowing off or being metabolised [15]. The kinetics of pyocyanin production was affected in a similar way in *mucA22* mutants, with a decrease relative to wildtype and suppressor in early stationary phase followed by continued production leading to 3 –fold higher levels than the wildtype and suppressor strain in late stationary phase cultures (Fig. 1B). Turning to elastase activity, levels were negligible in mid-log phase but throughout stationary phase the *mucA22* strains were deficient in elastase production compared to their parental strain (p<0.01 for each pair) (Fig. 1C), this being reversed in most of the suppressor strains. These findings indicate that *mucA22* mutation is associated with a global alteration of virulence factor regulation in stationary phase, although, the regulation of elastase production appears to differ from that of cyanide and pyocyanin.

### The *mucA22* mutation alters the expression of cyanide, pyocyanin and elastase biosynthetic genes

**Figure 2:**
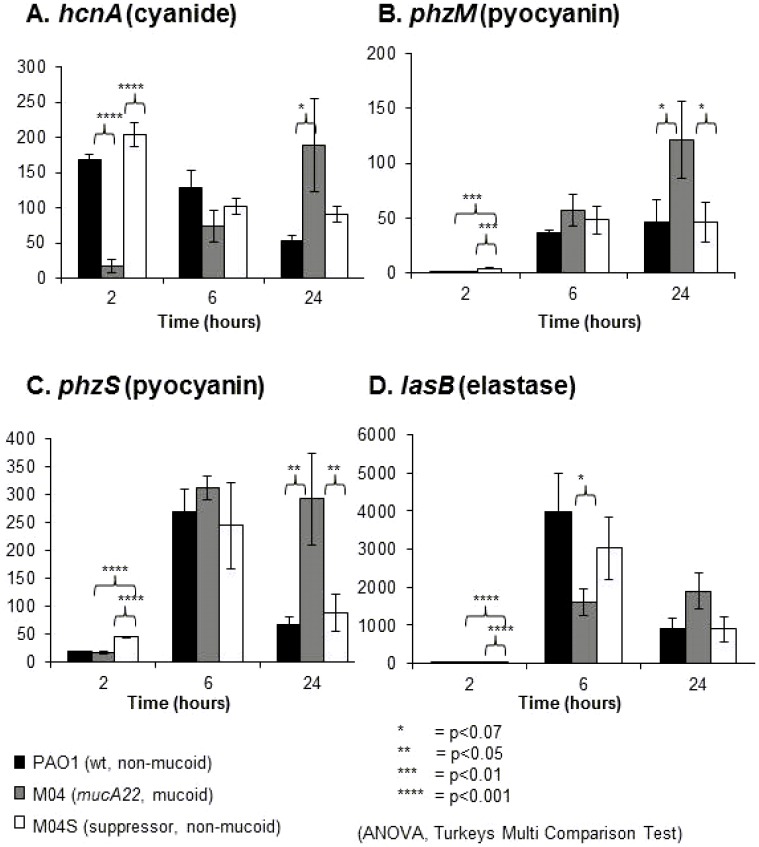
*mucA* mutation alters the expression of cyanide, pyocyanin and elastase genes. Expression profiles of *hcnA* (A), *phzM* (B), *phzS* (C) and *lasB*, (D) for PAO1 (wild type, non-mucoid) (black); M04 (*mucA22*, mucoid) (dark grey); and M04S (non-mucoid suppressor isolated from M04) (light grey). Values are averages of 3 biological replicates and the value for each replicate was the average of duplicate qRT-PCR technical replicates. Error bars are ±SEM. Data were analysed by ANOVA and post-hoc by Tukey's Multiple Comparison Test.

qRT-PCR was used to determine whether the phenotypic changes observed in *mucA22* mutants resulted from changes in gene expression. PAO1 and the suppressor strain M04S (Fig. 2A) showed similar *hcnA* expression levels to each other, with highest levels at 2 hours (mid-log phase) and 6 hours (early stationary phase) and decreased levels in late stationary phase (24 hours). It is interesting that significant *hcnA* expression is observed in the mid-log phase cultures at a time when no cyanide was detectable in the culture medium (Fig. 1), indicating that *hcnA* expression is induced in exponential phase well ahead of cyanide production in stationary phase. Expression in the *mucA22* mutant M04 was significantly lower at 2 and 6 hours and markedly increased at 24 hours. Overall, the pattern of *hcnA* expression is consistent with the cyanide production data presented in Fig. 1 (and Figs. S1 and S2) suggesting that the low-level cyanide production in early stationary phase followed by continued production in later stationary phase seen in *mucA22* strains results from differences in the expression of the cyanide synthase genes. *hcnA* transcripts were still evident in PAO1 at 24 hours even though cyanide levels in culture supernatant had dropped to zero (Fig. 2), suggesting that either post-transcriptional regulation is affecting cyanide production or it is being lost from culture as fast as it is being made.

The expression of *phzM* and *phzS* genes was very low in mid-log phase cultures, although lowest in *mucA22* strains (Fig. 2B and C), but was expressed at similar levels in early stationary phase cultures, suggesting that deceased pyocyanin production in *mucA22* strains may be a consequence of a delay in induction of pyocyanin gene expression (but this is not as marked as with the *hcnA* gene) or it may not be wholly explained by changes in gene expression. However, the *mucA22* strain M04 showed significantly increased expression of *phzM* and *phzS* compared to the wild type and suppressor strains at 24 hours, indicating that increased pyocyanin levels in late stationary phase cultures of mucoid strains correlated with increased pyocyanin gene expression.

*lasB* expression was detected only at very low levels in exponential phase cultures (2 h), but there was significantly increased *lasB* expression in all strains at 6 h and a significant difference between the wild type and *mucA22* strain (M04). At 24 h, all strains showed similar *lasB* expression.

The qRT-PCR gene expression data are broadly consistent with the phenotypic data and in particular show markedly increased levels of the *hcnA* and *phz* genes that are consistent with maintenance of increased cyanide and pyocyanin levels in late stationary phase.

### The *mucA22* mutation results in suppression of quorum sensing signal molecule production throughout growth

**Figure 3:**
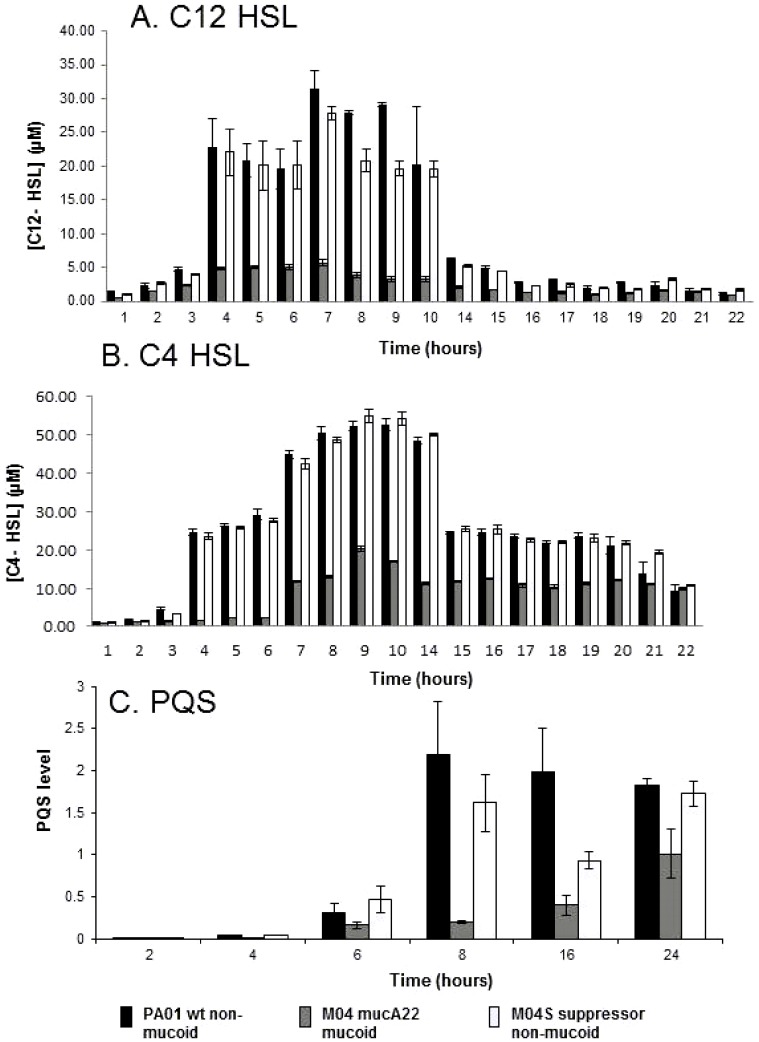
*mucA* mutation leads to suppression of quorum sensing signal molecule production throughout growth. Activities of the LasIR (A) RhlIR (B) and PQS (C) systems was compared in PAO1 (wild type, non-mucoid) (black), M04 (*mucA22*, mucoid) (dark grey) and M04S (non-mucoid suppressor isolated from MucA04) (light grey) using bioluminescent reporter strains to assay 3-oxo-C_12_ (lasIR) C_4_-HSL (RhlIR) and PQS signal molecule levels in supernatant. Bioluminescence was measured using a luminometer. Culture conditions were as described in Figure 1. Values are means of 3 independent replicates and error bars are ±SEM.

As cyanide, pyocyanin and elastase are quorum sensing-(QS)-regulated [38], we tested whether *mucA22* mutation led to a defect in the two N-acyl homoserine lactone (AHL)-dependent QS systems of *P. aeruginosa*; the *lasRI* and *rhlRI* systems that synthesise 3-oxo-C12-HSL and C4–HSL respectively [38]. The *mucA22* mutant M04 was greatly impaired in the production of both these AHL molecules (Fig. 3A and B; as were other independently isolated *mucA22* mutants of PAO1 and PAO381, Fig. S3). Wild type levels of both AHLs were observed in the non-mucoid suppressor strain.

The third quorum sensing system in *P. aeruginosa* is the 2-alkyl-4-quinolone (AQ or PQS)-dependent quorum sensing system (mediated by PQS; 2-heptyl-3-hydroxy-4-quinolone and HHQ; 2-heptyl-4-quinolone) and we found that the *mucA22* mutant M04 was also impaired in production of PQS (Fig. 3C). This was most apparent in early to mid-stationary phase (8 hours, t-test, p<0.05), whereas in late stationary phase (24 hours), the differences between wildtype and *mucA22* and the suppressor strain are less marked but still significant (t-test, p<0.05). These data are consistent with a quorum sensing defect being responsible for the changes in cyanide, pyocyanin and elastase production in early stationary phase cultures of mucoid strains.

**Figure 4:**
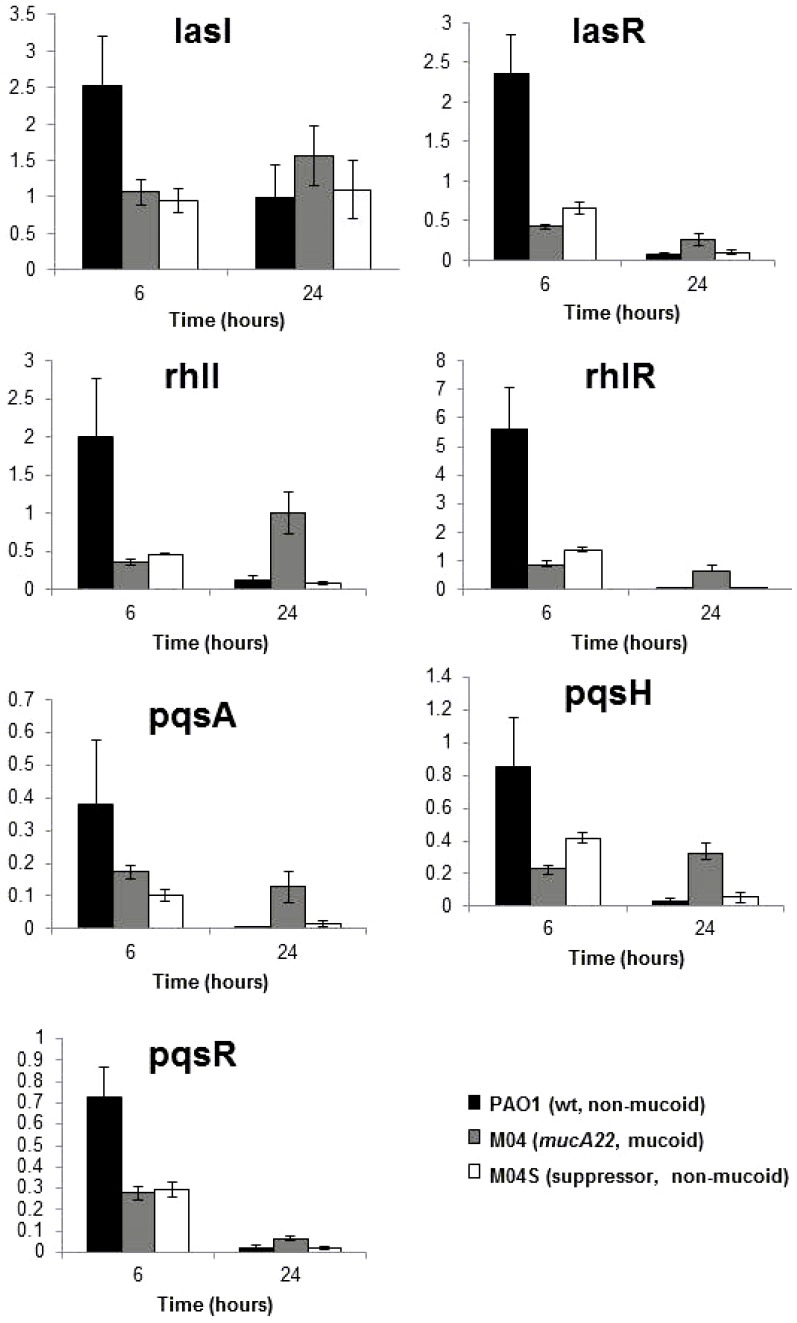
*mucA* mutation leads to suppression of quorum sensing gene expression. qRT-PCR expression profiles of quorum sensing regulatory genes for: PAO1 (wild type, non-mucoid) (black); M04 (*mucA22*, mucoid) (dark grey); and M04S (non-mucoid suppressor isolated from MucA04) (light grey). Values are averages of 3 biological replicates and the value for each replicate was the average of duplicate qRT-PCR technical replicates Error bars are ±SE and data were analysed by ANOVA and post-hoc by Tukey's Multiple Comparison Test.

We used qRT-PCR to determine the expression of *lasR*, *lasI* (2-oxo-C12-HSL system), *rhlR*, *rhlI* (C4-HSL system) and the *pqsA*, *pqsH* and *pqsR* genes (PQS system) (Fig. 4). In early stationary phase (6 h), M04 had a significant reduction in expression of AHL and PQS quorum sensing genes compared to the wild-type (Fig. 4), which suggested that reduced AHL and PQS levels in early stationary phase (Fig. 3) result from gene expression changes. However, quorum-sensing gene expression levels in the suppressor strain M04S was similar to M04 and did not reflect the levels of AHL and PQS molecules detected (Fig. 3); the reason for this is unclear. The correlation between the levels of quorum sensing gene expression and the AHL and PQS levels is less clear at 24 hours (Fig. 4).

We also tested whether adding back C4-HSL or 3-oxo-C12-HSL was able to recover the early stationary phase cyanide-production phenotype of *mucA22* strains. However, supplementing the culture medium with 100 µM C4-HSL or 3-oxo-C12 HSL did not recover early stationary phase (6–8 h) cyanide production in *mucA22* mutants (data not shown). However, as *rhlR* and *lasR* expression is repressed in *mucA22* strains (Fig. 4), it is likely that the levels of the RhlR and LasR proteins are too low to respond effectively to the added AHLs. We also considered the possibility that our *mucA22* mutants had acquired a *lasR* mutation, but PCR amplification and sequencing of the *lasR* gene from mutants showed none to be present.

These experiments demonstrate that the switch to mucoidy has a profound effect on the multiple quorum sensing systems of *P. aeruginosa*.

### *mucA22* mutation does not act through disruption of the *rsmY*/*rsmZ* regulatory network

Next we explored further how a defect in *mucA* could lead to a loss of quorum sensing and affect cyanide production. We reasoned that a defect in *mucA*, leading to increased expression of *algU* might act through disruption of *rsmY* and *rsmZ* regulatory systems, which through sequestration of the negative regulator of quorum sensing RsmA activate quorum sensing and cyanide production [39]–[43] and that this might occur through competition with an unidentified sigma factor that is a proposed component of the HptB -mediated region of this regulatory pathway [44]. To test this we introduced *rsmY*-and *rsmZ-lacZ* fusions into wild type (PAO1), *mucA22* mutant (M04) and suppressor strain (M04S) and measured β-galactosidase activity of reporter strains through growth into stationary phase. No significant differences in *rsmY*-and *rsmZ-lacZ* fusion expression were observed between wild type and *mucA22* strains at any stage during growth, indicating that expression *rsmY* and *rsmZ* is not compromised by *mucA22* mutation (Fig. S4).

### Plasmid expression of the cAMP-dependent transcription factor Vfr restores the quorum sensing defect and early stationary phase cyanide production in the *mucA22* mutant

**Figure 5:**
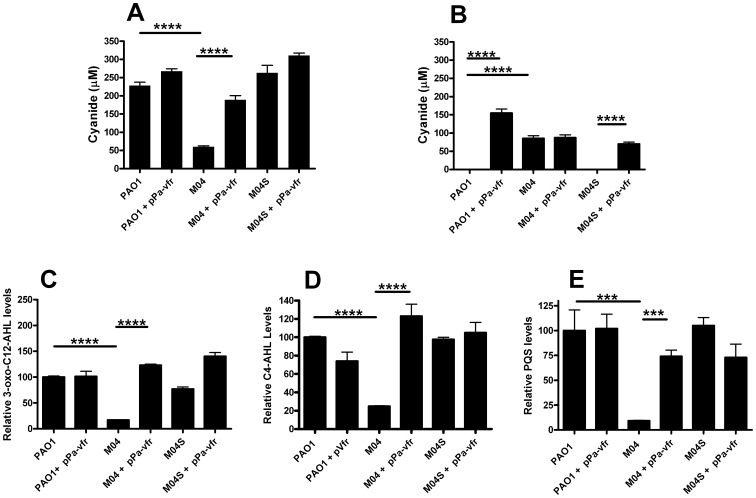
Plasmid expression of the cAMP-dependent transcription factor Vfr restores the early stationary phase cyanide production and the quorum sensing defect in the *mucA22* mutant. Cyanide concentration and QS molecule levels were measured in culture supernatants of wildtype (PAO1), *muc22* mutant (M04) and its suppressor strain (M04S) with or without the plasmid pPa-vfr. Cyanide levels after (A) 6 hours growth (early stationary phase) and (B) 24 hours growth (late stationary phase). (C) Relative 3-oxo-C12 AHL levels; (D) relative C4-AHL levels; (E) relative PQS levels after 8 hours growth (early stationary phase). Values are relative to the PAO1 8 hour value. All experiments are averages of three independent replicates error bars are ±SEM and data were analysed by ANOVA and post-hoc by Tukey's Multiple Comparison Test. **** p<0.001; *** p<0.01.

AlgU activates the expression of a number of genes, including the gene encoding the AlgR response regulator and together they activate the transcription of the alginate biosynthetic genes [45], [46]. The cAMP-dependent Vfr signalling system has been shown to be defective in *mucA* mutants at the level of *vfr* expression via a mechanism involves AlgU and AlgR [47]. Given that plasmid encoded Vfr restores cAMP-dependent Vfr signalling in a *mucA22* mutant [47], we investigated whether introduction of pPa-*vfr* into our isogenic strains resulted in the restoration of cyanide production. It is clear that cyanide production in early stationary phase is restored to wildtype levels in the *mucA22* mutant by pPa-*vfr* (Fig. 5A), suggesting that loss of Vfr contributes to the reduced peak cyanide production in *mucA22* mutants. The presence of pPa-*vfr* in wildtype and suppressor strains leads to them maintaining the production of cyanide into late stationary phase (24 h) in a way similar to M04, rather than the complete loss of cyanide production associated with the wildtype in the absence of pPa-*vfr* (Fig. 5B, Fig. 1). Furthermore, we found that plasmid borne expression of Vfr was able to restore 3-oxo-C12-HSL and C4–HSL and PQS levels to wildtype in 6 h stationary phase cultures of M04 (Fig. 5C–E).

## Discussion

The *mucA22* mutation is the predominant mutation leading to mucoidy in *P. aeruginosa* during chronic CF lung infections. In this present work we report that the switch to a mucoid phenotype via a *mucA22* mutation has a profound effect on stationary phase physiology. An important finding is that a persistent low-level production of cyanide and pyocyanin occurs in mucoid strains in late stationary phase compared to non-mucoid strains. We used spontaneous non-mucoid revertants of mucoid strains in this study due to the problematic nature of complementing or repairing *mucA22* mutations in the context of the unstable nature of the mucoid phenotype. The mucoid phenotype is frequently reported as unstable and non-mucoid variants emerge through suppressor mutations in culture and in the CF lung and where both phenotypes can coexist [36], [48]–[51]. Suppressor mutations in the *algU* gene have been described [51], but we did not find *algU* mutations in any of the suppressor stains used in this study and neither did they have identifiable mutations in known regulars of mucoidy including; *mucA*, *algD*, *algR*, and *lasR*.

Cody et al (2009) have reported the regulation of *hcnABC* genes by the AlgZ-AlgR two component regulatory systems and demonstrated the presence of HCN in cultures after 36 hours of growth [52]. This contrasts with our findings, which were highly reproducible in our hands. The only time we have seen cyanide in late stationary phase was using sealed flasks that prevent loss of cyanide gas by blowing off [15].Previously, mucoid CF isolates have been reported to be deficient in motility (due to inhibition of flagella production), pyocyanin production, elastase and total protease production, and decreased exotoxin A and exoenzyme S production as well as having reduced expression of type III secretion systems and type IV pili [26], [53]–[56]. Here we demonstrate that *mucA22* mutation and the consequent increased availability of the sigma factor AlgU leads to a more complex and subtle change in the stationary phase regulation of virulence factor production than previously recognised (Figs. 1, 2 and S1). We show that *mucA22* mutation results in repression of AHL- and AQ- dependent quorum sensing systems (Figs. 3 and 4) leading to decreased production of the virulence factors cyanide, pyocyanin and elastase in early stationary phase, but also changes the regulation of cyanide and pyocyanin in late stationary phase leading to their continued production in non-growing cultures which is in stark contrast to non-mucoid strains (Fig. 1). qRT-PCR data indicates that *mucA22* mutation leads to changes in the regulation of expression of the HCN synthase genes (*hcnABC*), the *phz* genes involved in pyocyanin synthesis and the elastase encoding *lasB* gene (Fig. 2). The effects of *mucA* mutation on stationary phase properties that we observe fit with the recent finding that MucA modulates osmotic stress tolerance in stationary phase, but not exponential phase, cultures of *P. aeruginosa* [29].

Previous publications provide contradictory findings regarding cyanide production in mucoid and non-mucoid strains. Carterson et al found that mucoid strains produced seven times **more** cyanide than non-mucoid strains [57]. This is in contrast with a study [58] that found a **decrease** in expression of the *hcnABC* genes in mucoid *P. aeruginosa*, and a recent survey of hydrogen cyanide (HCN) released into the gas phase by 96 genotyped *P. aeruginosa* strains that found reduced cyanide accumulation by mucoid strains [59]. The apparent incongruous nature of these reports is explained by our data. While Carterson *et al* [57] measured cyanide given off from plate grown cultures (most cells of which will have reached stationary phase), Rau *et al* [58] performed a transcriptomic study on mid-exponential phase cultures. Our data shows that in exponential phase cultures the *hcnA* gene is expressed in non-mucoid strains (in advance of cyanide being detectable in the growth medium), but there is a 10-fold lower level of expression in *mucA22* mucoid strains, which is consistent with data of Rau *et al* [58]. This differential gene expression is maintained into early stationary phase when cyanide levels peak at a 2.5-fold lower level in *mucA* strains (Fig. 1 and 2). There is complete loss of cyanide production in non-mucoid strains in late stationary phase (Fig. 1), whereas mucoid strains maintain cyanide production, which explains, we suggest, the high levels of cyanide trapped from the plate grown cultures by Carterson *et al* [57]. Indeed when we assayed cyanide in our strains by the same plate assay method we obtained a similar result to Carterson *et al* (2004) (data not shown). In an earlier study, Firoved and Deretic [25] reported an increase in expression of *lasB*, *hcnA* and *hcnC* in a *mucA* strain, grown to mid-exponential phase, compared to its non-mucoid parent, but cyanide levels were not measured. These data are different to both our findings and those of Rau et al [58], and are not easily explained other than by possible growth condition and strain differences.

Elastase peak levels were also reduced in *mucA22* mutants, but *lasB* showed different regulation in late stationary phase to cyanide and pyocyanin, indicating operation of a different regulatory mechanism.

As cyanide, pyocyanin and elastase are all positively regulated by quorum sensing we tested whether the change in their regulation in a *mucA22* mutant might be through an effect on quorum sensing. We found that while *mucA22* strains showed induction of LasR/3-oxo-C12-HSL, RhlR/C4-HSL and AQ-dependent quorum sensing systems their levels were reduced to approximately 17%, 35%, and 10% respectively of wild type levels at the time of peak production in early stationary phase (Fig. 3), which can explain the decreased cyanide, pyocyanin and *lasB* expression in early stationary phase in *mucA* strains. In late stationary phase (22 to 24 hours) *mucA* strains have comparable or lower levels of QS molecules compared to wild type and suppressor strains, and so the maintenance of cyanide and pyocyanin production at these time points must be dependent on other regulatory factors. Morici et al have previously demonstrated a link between the AlgR response regulator (a positive regulator of *hcnABC*) and the RhlR/C4-HSL quorum sensing system [28]. They showed by both transcriptomic and gene fusion studies that AlgR partly suppressed the RhlR/C4-HSL quorum sensing system, but in a biofilm specific manner; it did not affect planktonic cultures. Rau et al [58] reported an effect of *mucA* mutation on the RhlR/C4-AHL quorum sensing system, showing decreased *rhlR* and *rhlI* expression at a single time point in mid-exponential phase cultures and also decreased C4-HSL production when cultures reached an OD_600_ of 1.0. Our study has confirmed and extended these findings through the growth cycle from early exponential phase to late stationary phase and to effects on the *lasR* and PQS quorum sensing systems.

**Figure 6:**
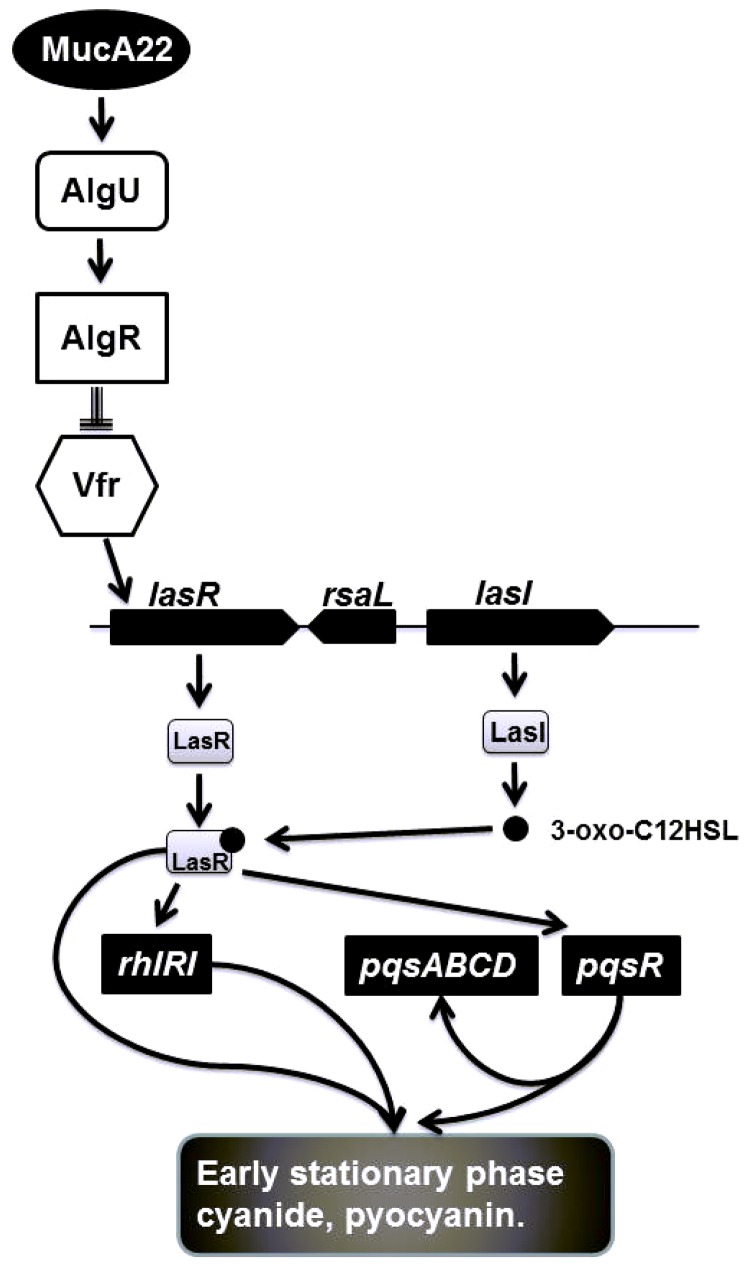
Model to explain early stationary phase down regulation of cyanide, pyocyanin and elastase production by mucA22 mutation of *P. aeruginosa*. This figure is an adaptation and extension of Figure 7 from [47]. Solid arrows indicate positive regulation and T-bar negative regulation. *MucA22* mutation results in release of AlgU, which is then free to associate with core RNA polymerase and direct expression from its target genes, which include the gene for the transcriptional regulator AlgR. AlgR is a repressor of the cAMP-dependent transcriptional regulator, VfR, which is a positive regulator of the LasRI quorum sensing system. AlgR inhibition Vfr transcription will result in down regulation of the LasRI quorum sensing system, which since it is the dominant QS system will act to down regulate both the RhlRI and AQ (PQS) QS systems, resulting in suppression of the entire QS network and decreased expression of virulence factors in early stationary phase.

We next addressed how the *mucA22* mutation could lead to such marked effects on quorum sensing? We found no evidence for a role for the regulatory small RNAs *rsmY* and *rsmZ* in this regulation, but we did find data supporting a role for Vfr, the cAMP-dependent positive regulator of the Las quorum sensing system (Fig. 5). Vfr has recently been shown to be down regulated in *mucA* strains via an AlgR dependant mechanism [47] and we found that plasmid expression of Vfr restored the quorum sensing defect in *mucA22* mutants and consequently restored cyanide production. So while at this stage a complete mechanism to explain our findings is not deducible, a testable model building on these findings is shown in Fig. 6.

The switch to a mucoid phenotype is one of the most frequently encountered and clinically significant changes undergone by *P. aeruginosa* and occurs during chronic infection of the cystic fibrosis lung. It occurs in most CF lung infections and is concurrent with a decrease in the clinical condition of the patient [2], [18]. Evidence indicates that alginate provides *P. aeruginosa* with protection from the host immune response by inhibiting phagocytosis, protecting against the oxidative burst and by providing an immunomodulatory role and so provides a selective advantage in the lung [60], [61]. However the pleiotropic effects of *mucA22* mutation on quorum sensing and virulence factor regulation may also provide a powerful selection pressure for the mucoid switch. Genetic analysis of *P. aeruginosa* isolates from the airways of multiple CF patients has revealed that one of the most common targets of mutation in CF isolates is the *lasR* gene [62], suggesting a strong selection for the loss of *lasR* function and subsequent down-regulation of quorum sensing pathways in the CF airways. Our data indicates that the switch to mucoidy provides a second route for down regulation of quorum sensing pathways, which could provide a selection pressure for the switch to mucoidy during CF lung infections. Whether these findings are of significance *in vivo* requires further study in appropriate model systems and of clinical strains.

If this mechanism was reproduced in vivo during CF lung infection it would lead to continued production and cyanide and pyocyanin in non-growing populations of *P. aeruginosa*, which could be significant as these compounds can damage lung tissue and provide a source of nutrients for growth and survival. While cyanide is only synthesised aerobically HCN made by P. aeruginosa in oxic regions of the lung that diffuse into anoxic areas would potentially inhibit anaerobic respiration in the absence of protective mechanism against HCN [16], [63].

## Supporting Information

Figure S1 ***mucA22*** **, mucoid strains are deficient in cyanide, pyocyanin and elastase production in early stationary phase.** Cyanide (**A**), Pyocyanin (**B**) and Elastase (**C**) in supernatant of liquid cultures of non-mucoid wild type (black), mucoid *mucA22* (dark grey) and non-mucoid suppressor (light grey) strains at 2 hours post stationary phase initiation. Cultures were incubated in 50 ml LB in 250 ml conical flasks in orbital shaker at 200 rpm and 37°C and grown until 2 hour post stationary phase initiation (6 hours in all cases); growth was followed by determining OD_600_ every hour. Cyanide concentrations were measured with an ion selective micro electrode. Pyocyanin levels were determined by chloroform/HCl extraction followed by absorbance measurement 520 nm. Elastase levels were determined by assaying enzymatic breakdown of Elastin-Congo red then measurement of liberated Congo red in a spectrophotometer at A_495_. Values are means of 3 independent replicates and error bars are ±SEM.
(TIF)

Figure S2 **Growth and Cyanide production for PA0381 and PAO578 (** ***mucA22*** **).** Optical density on primary y axis, (solid markers) cyanide concentration on secondary y axis, (empty markers) for (A) PAO381 (wild type, non-mucoid) (black); and PAO578 (*mucA22*, mucoid derivative of PAO381) (dark grey).
(TIF)

Figure S3 ***mucA*** **mutation leads to suppression of quorum sensing signal molecule production in independently isolated** ***mucA22*** **mutants isolated in PAO1 and PAO381 backgrounds.** Activities of the C4-AHL (A) and 3-oxo-C12-AHL (B) was compared in wild type, non-mucoid strains (WT), *mucA22* (MUCOID) and suppressor strains after 2 hours (mid-log) and 6 hours (early stationary phase) of growth in LB medium. Values are means of 3 independent replicates and error bars are ±SEM.
(TIF)

Figure S4 ***mucA22*** **mutation does not act through disruption of the** ***rsmY*** **/** ***rsmZ*** **regulatory network.** The wildtype strain PAO1, the *mucA22* mutant M04 and its suppressor strain M04S carrying the *rsmY-lacZ* or *rsmZ-lacZ* gene fusions [44] were grown in LB and growth followed (A) and samples assayed for β-galactosidase (B), the values plotted being ±SEM (n = 3 biological replicates).
(TIF)

Table S1 **Bacterial strains and plasmids.**
(DOC)
