# Supplementary figures and images for: The Mucoid Switch in Pseudomonas aeruginosa Represses Quorum Sensing Systems and Leads to Complex Changes to Stationary Phase Virulence Factor Regulation
Source: PLoS One. 2014 May 22;9(5):e96166. doi: 10.1371/journal.pone.0096166 (PMC4031085; doi:10.1371/journal.pone.0096166)

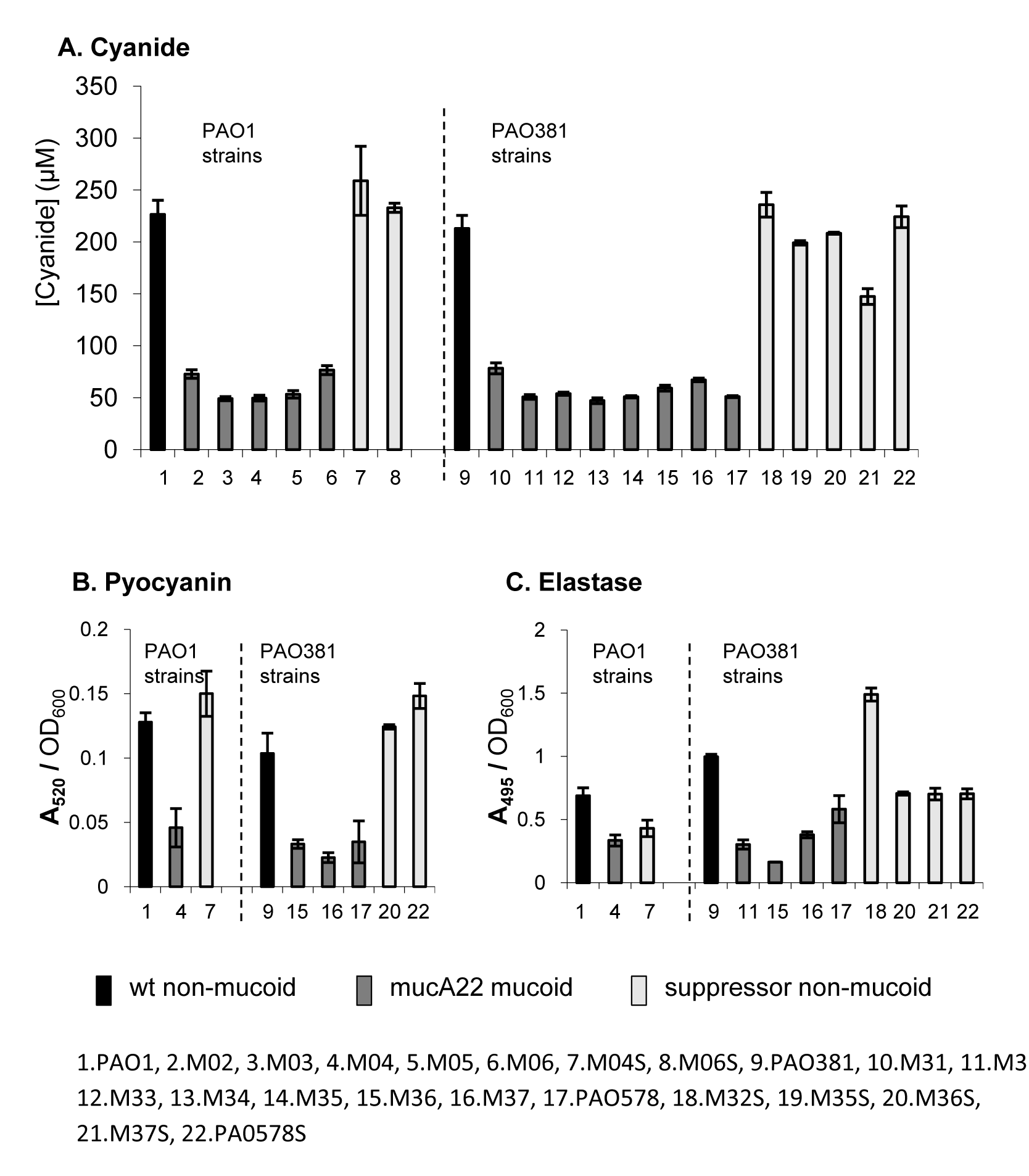

Supplement: Figure S1 — mucA22 , mucoid strains are deficient in cyanide, pyocyanin and elastase production in early stationary phase. Cyanide (A), Pyocyanin (B) and Elastase (C) in supernatant of liquid cultures of non-mucoid wild type (black), mucoid mucA22 (dark grey) and non-mucoid suppressor (light grey) strains at 2 hours post stationary phase initiation. Cultures were incubated in 50 ml LB in 250 ml conical flasks in orbital shaker at 200 rpm and 37°C and grown until 2 hour post stationary phase initiation (6 hours in all cases); growth was followed by determining OD600 every hour. Cyanide concentrations were measured with an ion selective micro electrode. Pyocyanin levels were determined by chloroform/HCl extraction followed by absorbance measurement 520 nm. Elastase levels were determined by assaying enzymatic breakdown of Elastin-Congo red then measurement of liberated Congo red in a spectrophotometer at A495. Values are means of 3 independent replicates and error bars are ±SEM. (TIF) [file pone.0096166.s001.tif]

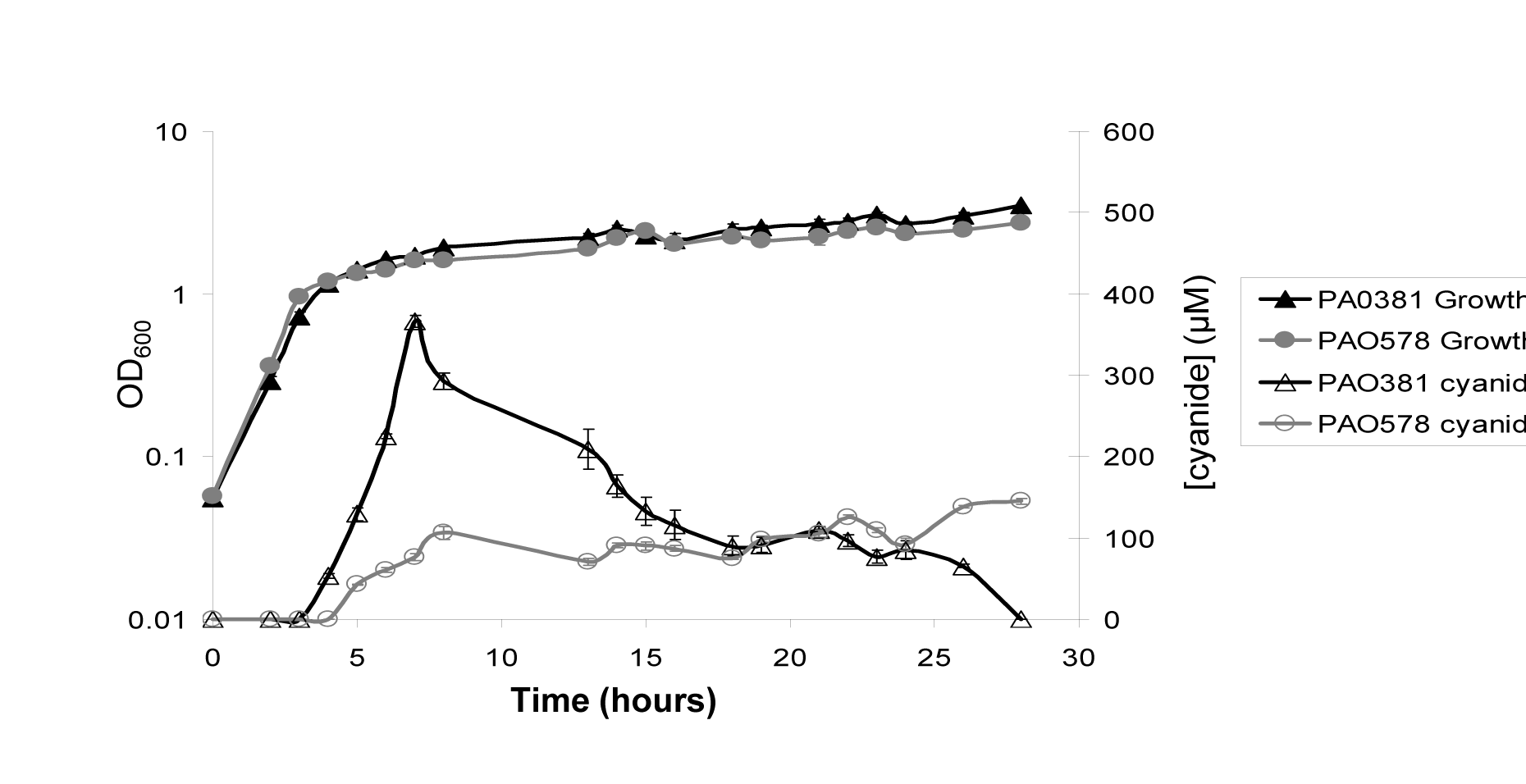

Supplement: Figure S2 — Growth and Cyanide production for PA0381 and PAO578 ( mucA22 ). Optical density on primary y axis, (solid markers) cyanide concentration on secondary y axis, (empty markers) for (A) PAO381 (wild type, non-mucoid) (black); and PAO578 (mucA22, mucoid derivative of PAO381) (dark grey). (TIF) [file pone.0096166.s002.tif]

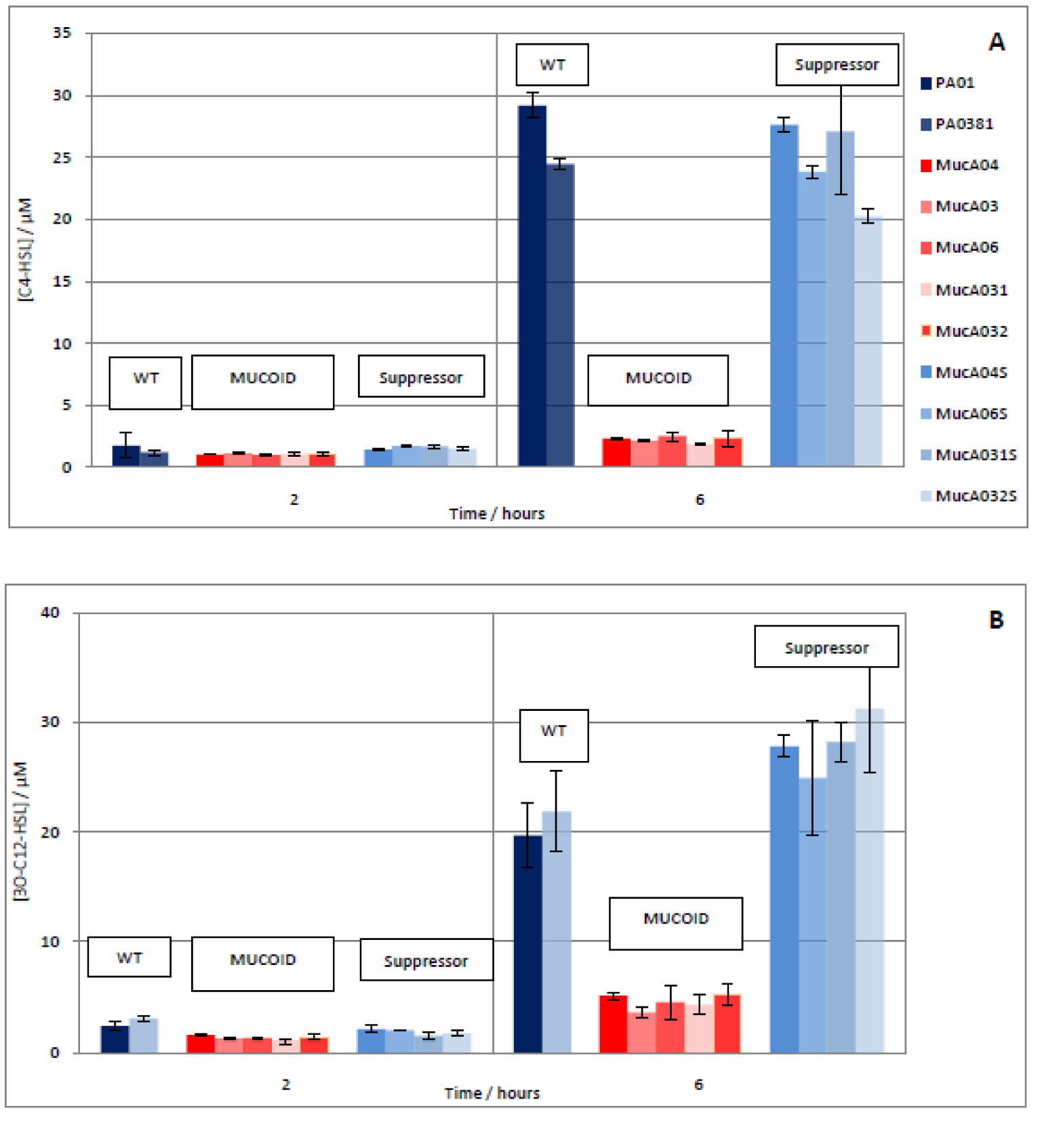

Supplement: Figure S3 — mucA mutation leads to suppression of quorum sensing signal molecule production in independently isolated mucA22 mutants isolated in PAO1 and PAO381 backgrounds. Activities of the C4-AHL (A) and 3-oxo-C12-AHL (B) was compared in wild type, non-mucoid strains (WT), mucA22 (MUCOID) and suppressor strains after 2 hours (mid-log) and 6 hours (early stationary phase) of growth in LB medium. Values are means of 3 independent replicates and error bars are ±SEM. (TIF) [file pone.0096166.s003.tif]

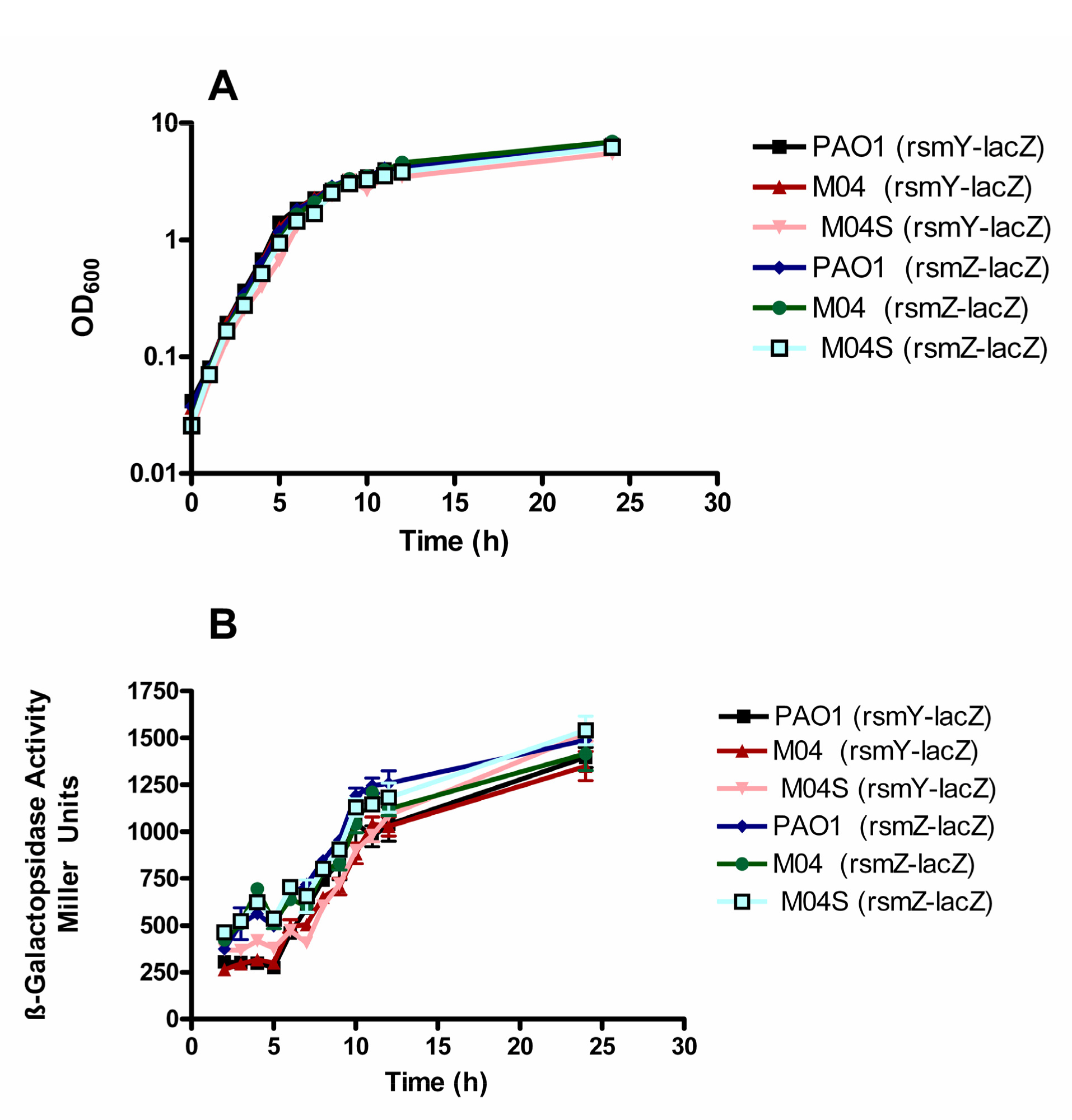

Supplement: Figure S4 — mucA22 mutation does not act through disruption of the rsmY / rsmZ regulatory network. The wildtype strain PAO1, the mucA22 mutant M04 and its suppressor strain M04S carrying the rsmY-lacZ or rsmZ-lacZ gene fusions [44] were grown in LB and growth followed (A) and samples assayed for β-galactosidase (B), the values plotted being ±SEM (n = 3 biological replicates). (TIF) [file pone.0096166.s004.tif]
