# Supplementary material for: The Mucoid Switch in Pseudomonas aeruginosa Represses Quorum Sensing Systems and Leads to Complex Changes to Stationary Phase Virulence Factor Regulation
Source: PLoS One. 2014 May 22;9(5):e96166. doi: 10.1371/journal.pone.0096166 (PMC4031085; doi:10.1371/journal.pone.0096166)
Supplement: Table S1 — Bacterial strains and plasmids. (DOC) [file pone.0096166.s005.doc]

Table S1 Bacterial strains and plasmids

| Strain / plasmid | Relevant information | Source / (reference) |
| --- | --- | --- |
|  |  |  |
| P. aeruginosa |  |  |
| PAO1 | Wild-type, prototrophic strain, clinical isolate | Lab strain, original source - B. Holloway |
| PAO381 | *leu*-38, *str*-2. PAO1 derivative, non-mucoid. | J. Govan |
| PAO578 | *leu*-38, *str*-2, *mucA-22*. Mucoid PAO381 derivative. | J. Govan |
| PAO578S | Non mucoid. Spontaneous PAO578 mucoid suppressor mutant. | Isolated in this laboratory |
| PAO579 | *leu*-38, *str*-2, *muc23*. Mucoid PA0381 derivative, *mucA22* independent. | J. Govan |
| M02-06 | *mucA22*, mucoid PAO1 derivative | This study |
|  |  |  |
| M31-35 | *mucA22*, mucoid PAO381 derivative | This study |
| M04S,06S | Non mucoid suppressors, derivatives of M04,06 | This study |
|  |  |  |
| M31S,32S,35S | Non mucoid suppressors, derivatives of M31,32,35 | This study |
| PAO1ΔpqsA pqsA::CTXlux | PAO1 *pqsA* mutant with chromosomally integrated  pqsA´::luxCDABE fusion | [2,3,4] |
| E. coli |  |  |
| DH5α | recA, gyrA |  |
| S17.1 | *recA, pro* (2-TET::mu Kan::Tn7) |  |
| Plasmids |  |  |
| pJQ200KS | *sacB*, *bla, oriT ,oriV, lacZα* +ve selection suicide vector, Gm^R^, Suc^s^ |  |
| pBR002 | *mucA22* delivery vector. 1 kb fragment containing *mucA22* from PAO578 in the NotI site of pJQ200KS, Gm^R^ | This study |
| pSB401 | AHL biosensor luxR luxI (Photobacterium fischeri [ATCC 7744])::luxCDABE (Photorhabdus  luminescens [ATCC 29999]) fusion; pACYC184 derived, Tc^R^ | [2] |
| pSB536 | *ahyR*_ *ahyI*::*luxCDABE*; Amp^R^, ColE1 origin | [2] |

1. Fyfe JA, Govan JR (1980) Alginate synthesis in mucoid *Pseudomonas aeruginosa*: a chromosomal locus involved in control. J Gen Microbiol 119: 443-450.

2. Fletcher MP, Diggle SP, Camara M, Williams P (2007) Biosensor-based assays for PQS, HHQ and related 2-alkyl-4-quinolone quorum sensing signal molecules. Nat Protoc 2: 1254-1262.

3. Fletcher MP, Diggle SP, Crusz SA, Chhabra SR, Camara M, et al. (2007) A dual biosensor for 2-alkyl-4-quinolone quorum-sensing signal molecules. Environ Microbiol 9: 2683-2693.

4. Diggle SP, Matthijs S, Wright VJ, Fletcher MP, Chhabra SR, et al. (2007) The *Pseudomonas aeruginosa* 4-quinolone signal molecules HHQ and PQS play multifunctional roles in quorum sensing and iron entrapment. Chemistry & Biology 14: 87-96.

5. Quandt J, Hynes MF (1993) Versatile suicide vectors which allow direct selection for gene replacement in Gram-negative bacteria. Gene 127: 15-21.
